# Free gracilis end-to-side microanastomosis to a peronea arteria magna: a case report

**DOI:** 10.1186/s13256-021-03133-5

**Published:** 2021-12-13

**Authors:** Dallan Dargan, Raghuram Lakshminarayan, Cher Bing Chuo

**Affiliations:** 1 Department of Plastic Surgery, Hull University Teaching Hospitals NHS Trust, Hull, UK; 2 Department of Radiology, Hull University Teaching Hospitals NHS Trust, Hull, UK

**Keywords:** Free tissue transfer, Peronea arteria magna, Lower limb trauma, Open fracture, Polytrauma, Anatomical variant, Case report

## Abstract

**Background:**

Complex orthoplastic lower limb trauma in individuals with multiple injuries requires considerable resources and interdisciplinary collaboration for good outcomes. We present the first reported end-to-side free flap microanastomosis for lower limb trauma reconstruction involving a peronea arteria magna without radiographic collaterals.

**Case presentation:**

A 55-year-old Caucasian gentleman involved in road traffic collision sustained an open tibial fracture on the anteromedial distal third of the left lower leg with local degloving and a subtotal right foot and ankle degloving. Both injuries were reconstructed with free tissue transfer. A left lower limb peronea arteria magna successfully received a free gracilis muscle flap by end-to-side microanastomosis and perfusion of the foot was preserved. This rare anatomical variant and its anatomy is reviewed, as well as a description of the suggested preoperative planning and technique for reconstruction.

**Conclusions:**

Successful free flap reconstruction may be performed to a lower limb with a peronea arteria magna recipient as the lone vessel supplying the foot in trauma, although preoperative counseling of the risks, benefits, and options are essential.

**Level of evidence:**

Level V, case report

## Introduction

Lower limb open fractures requiring free flap reconstruction in polytrauma provide a reconstructive challenge when arterial anatomical variations are encountered. Contrast enhanced vascular imaging improves preoperative planning; however, the anatomy of the usual recipient vessels (anterior or posterior tibial artery) may vary considerably. The presence of a rare pattern of arterial distribution in the lower limb, called peronea arteria magna (PAM), where the peroneal artery is the lone arterial supply to the foot, may reduce the free tissue reconstructive options considerably. A systematic review of over 5000 limbs reports a dominant peroneal artery in 5.2% and a PAM in 0.4% [1].

The unified classification of infrapopliteal arterial branching patterns [2] modified from Lippert *et al.* [3], outlines a series of variations that reconstructive surgeons may utilize when planning free tissue transfer procedures (Table 1). The cost of devascularizing complications of surgery related to unrecognized PAM in free fibular harvest has been estimated as $170,000 USD, versus a standard free flap with no complications at $30,000 USD, which has been used as an argument for routine preoperative magnetic resonance angiography and traditional angiography [4].

**Table 1:** Kim–Lippert classification of the infrapopliteal arterial branching variations

Type I	Normal level of branching (proximal to the lower border of the popliteus muscle)
IA	The PA divides into the AT, the TPT (later divides into PT and PR)
IB	The PA trifurcates into AT, PT, and PR
IC	The PA divides into the PT, the anterior TPT (later divides into AT and PR)
Type II	High division of the PA (at or above the knee joint)
IIA	The AT originates at or above the knee joint
IIB	The PT originates at or above the knee joint
IIC	The PR originates at or above the knee joint
Type III	Hypoplastic or aplastic branching with altered distal supply.
IIIA	Hypoplastic/aplastic PT
IIIB	Hypoplastic/aplastic AT
IIIC	Both tibial arteries are hypoplastic/aplastic (PAM)

*PA* popliteal artery,*AT* anterior tibial artery, *TPT* tibioperoneal trunk, *PT* posterior tibial artery, *PR* peroneal artery, *PAM* peronea arteria magna. Reproduced with permission, Abou-Foul and Borumandi, Microsurgery, 2016. Oxford University Press [4]

### Case report

A 55-year-old Caucasian male cyclist was brought to a major trauma center by ambulance after he was involved in an alleged unwitnessed collision with a heavy goods vehicle. He was found on the road at 05:30, time of impact unknown.

The individual was right-handed and employed as a fish filleter. He was fully ambulant and independent for activities of daily living prior to admission. A bicycle or motorcycle were his main means of transportation. He lived with his wife in a house with internal stairs and external steps, a downstairs toilet, and upstairs bedroom. A family history of alcohol misuse was noted but no other familial medical conditions recorded.

Past medical history included diverticulosis, right bundle branch block, and a past closed right distal third tibia and fibula fracture 27 years earlier, which was successfully managed non-surgically. No regular medications were taken prior to admission and the gentleman was a non-smoker who consumed no alcohol.

In the ambulance, he was given intravenous co-amoxiclav 1.2 g, tranexamic acid 1 g, ketamine 2 mg/kg, paracetamol 1 g, ondansetron 4 mg, and intramuscular (IM) tetanus toxoid. On arrival, he was hypotensive with a blood pressure of 71/41 mmHg, a heart rate of 60 beats per minute, hypothermic with a temperature of 33.9 °C, respiratory rate of 20 breaths per minute, and oxygen saturation of 99% on high-flow oxygen by non-rebreather mask.

**Fig. 1:**
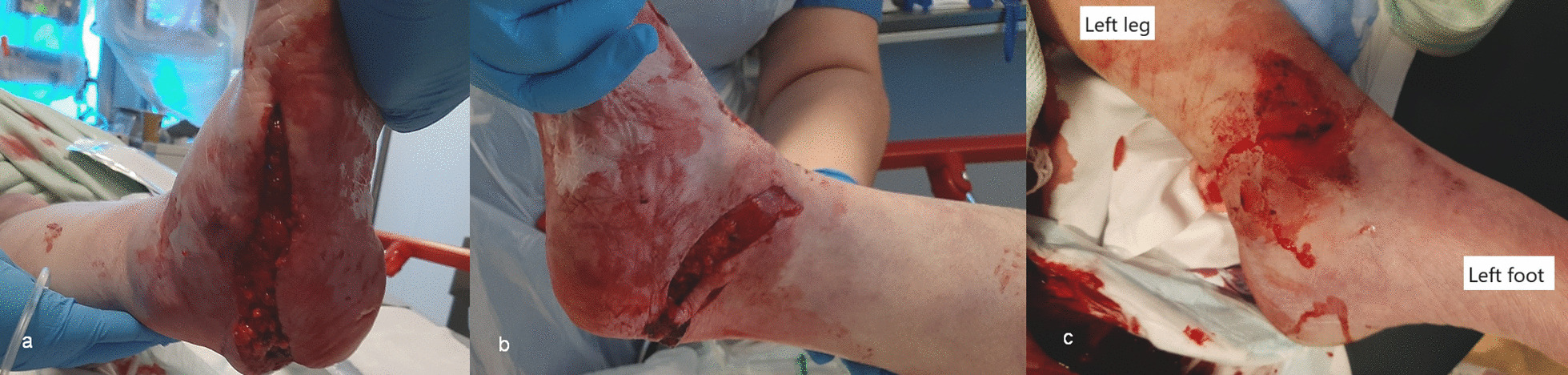
**a**–**c** Clinical images of the right foot and ankle (**a** and **b**) showing the degloving injury to the heel pad, the relative pallor of the foot and toes, and left ankle (**c**) showing the wound with surrounding devitalised skin overlying the left distal tibia

Physical examination revealed cold, pale skin, both centrally and peripherally, dry mucous membranes, and his airway was clear with no obvious chest injury. There was bleeding from both ankle wounds and a right arm wound. Clinical deformity of both ankles and the right upper limb were present. He had subtotal degloving of the right sole from the plantar heel pad to mid-foot, with a closed medial malleolar fracture suitable for non-surgical management (Fig. 1a–b). On the left, there was an anteromedial wound over the distal third of the leg overlying a Weber C bimalleolar fracture (Fig. 1c). The right foot was initially hypoperfused (capillary refill time of 3–4 seconds, no palpable pulses), and the left foot well perfused (capillary refill time < 2 seconds, palpable dorsalis pedis pulse). His neurological evaluation identified a Glasgow Coma Scale of 15/15 and he was noted to be agitated. He had abrasions to his scalp and bruising to his forehead and right cheek. He was diagnosed with a right humerus fracture and radial nerve palsy, however no other sensory deficit was identified.

Computed tomography (CT) of the head, neck, chest, abdomen, and pelvis excluded other injuries. The right foot perfusion improved to a capillary refill time of 2 seconds after fluid resuscitation with two units of packed red cells and 1.5 L crystalloids and application of plaster of Paris splints to both ankles and right upper limb. Laboratory findings after transfusion showed anemia and leukocytosis consistent with blood loss and acute phase response. Hemoglobin was 121 g/L (135–175 g/L), white blood cell count 26.4 × 10^9^/L (4.0–11.0 × 10^9^/L), and platelet count 289 × 10^9^/L (150–400 × 10^9^/L). Serum biochemical indices and liver function tests were within reference range, except creatinine 125 µmol/L (65–114 µmol/L), bicarbonate 19 mmol/L (24–32 mmol/L), and albumin 30 g/L (36–48 g/L). Coagulation parameters (prothrombin and activated partial thromboplastin time) were within reference ranges. Urinalysis at admission was not recorded.

Debridement of the lower limb wounds and application of Hoffman external fixation (Stryker, Kalamazoo, MI, USA) to the left lower limb fracture was performed the following day, together with internal fixation of the humeral shaft fracture. The left ankle wound was 8 × 5 cm, communicated directly with the tibial fracture, and was associated with localized subcutaneous degloving towards the Achilles tendon. A topical negative pressure wound therapy (TNPWT) dressing was applied to this Gustilo–Anderson Grade 3b open fracture after debridement.

There was multi-planar degloving of the right heel pad involving 60% of the plantar skin (25 × 27 cm), which appeared perfused (capillary refill 2 seconds), communicating with additional degloving of the dorsal foot where there was a 5 cm diameter wound on the dorsolateral foot and a 6 × 7 cm wound on the central dorsum of foot with exposed extensor tendons. Extensive contamination of the foot degloving was debrided. The right foot wound edges were loosely apposed with sutures, and TNPWT was applied enclosing the foot.

Following admission, intravenous co-amoxiclav 1.2 g TDS was continued for 10 days, and intravenous metronidazole 500 mg BD was added on days 7–10 because of positive culture of mixed coliforms and fecal microorganisms from right heel wound swabs.

Analgesia comprised paracetamol 1 g QDS, oxycodone modified release 10 mg BD, supplemented with oxycodone immediate release 5–10 mg PRN. Dalteparin 5000 units subcutaneously OD was administered as thromboprophylaxis. An oral (PO) phosphate supplement was administered for 14 days for hypophosphatemia commencing on the third day of admission.

**Fig. 2:**
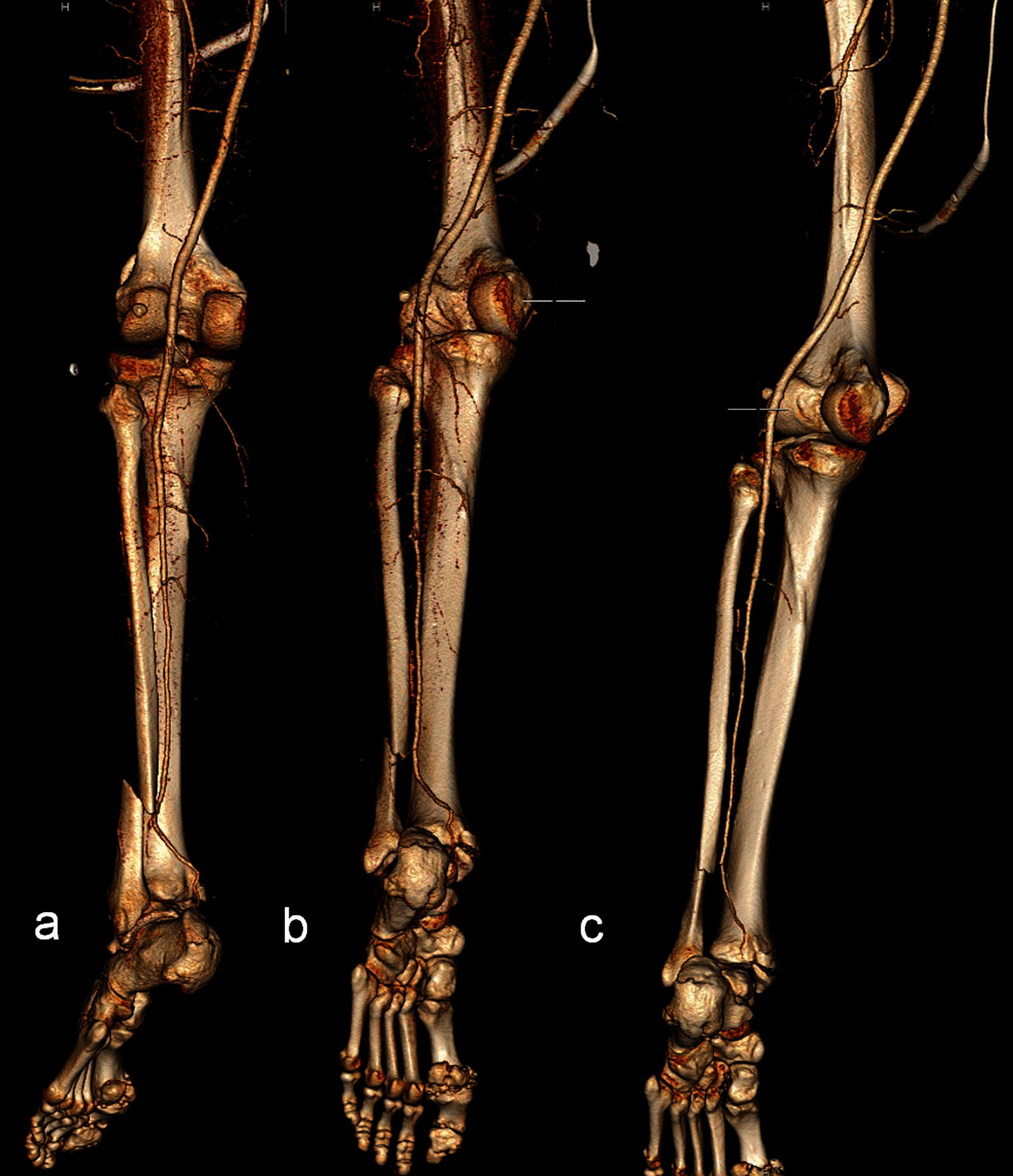
**a**–**c** Images from a three-dimensional reconstruction series of left lower limb CT angiogram, demonstrating the absence of both tibial arteries and course of the PAM along the usual course of the peroneal artery proximally, then the PAM courses posterior to the distal tibia and then behind the medial malleolus where the posterior tibial artery is typically located, and a bimalleolar ankle fracture. **a** Posterior view with internal rotation, **b** posterior view, **c** posterior view with external rotation

**Fig. 3:**
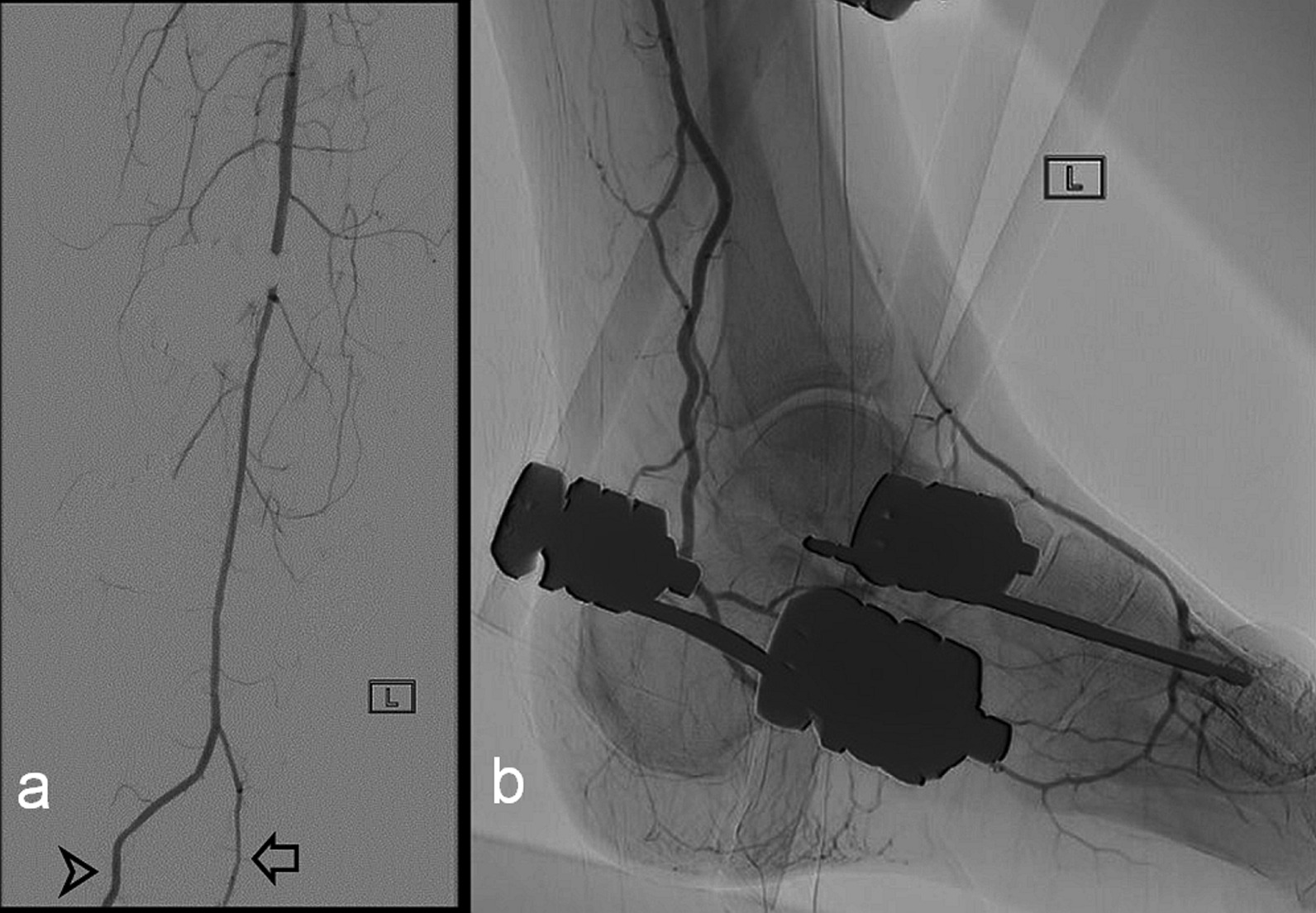
**a** and **b** Images from a percutaneous angiogram of the left lower limb. **a** Anteroposterior digitally subtracted imaging of the leg distal to the popliteal artery demonstrating a single below-knee dominant vessel (PAM), with a small branch as continuation of the usual course of the peroneal artery (arrow) and site of subsequent end-to-side microanastomosis to the PAM (arrowhead). **b** Lateral image of the left ankle showing the PAM passing posterior to the medial malleolus and into the plantar arch, with retrograde flow into the dorsalis pedis artery, but no apparent contrast in the region of the anterior tibial artery. An external fixator is present

A CT angiogram of the lower limbs was performed to assess the arterial contributions to both feet. This identified an unexpected finding of a left peronea arteria magna (PAM) (Fig. 2a–c), and normal three vessel arterial flow to the right foot. Percutaneous angiography was recommended to fully evaluate left foot perfusion, which revealed that the PAM supplied the plantar arch. The anterior and posterior tibial arteries were hypoplastic or aplastic and appeared to provide no contribution to the plantar arch (Fig. 3a and b).

Preoperative patient counseling was informed by the CT angiogram and angiography findings, which helped us to specifically explain the risks of acute ischemia or steal phenomenon following end-to-side microanastomosis to the only artery to the left foot.

**Fig. 4:**
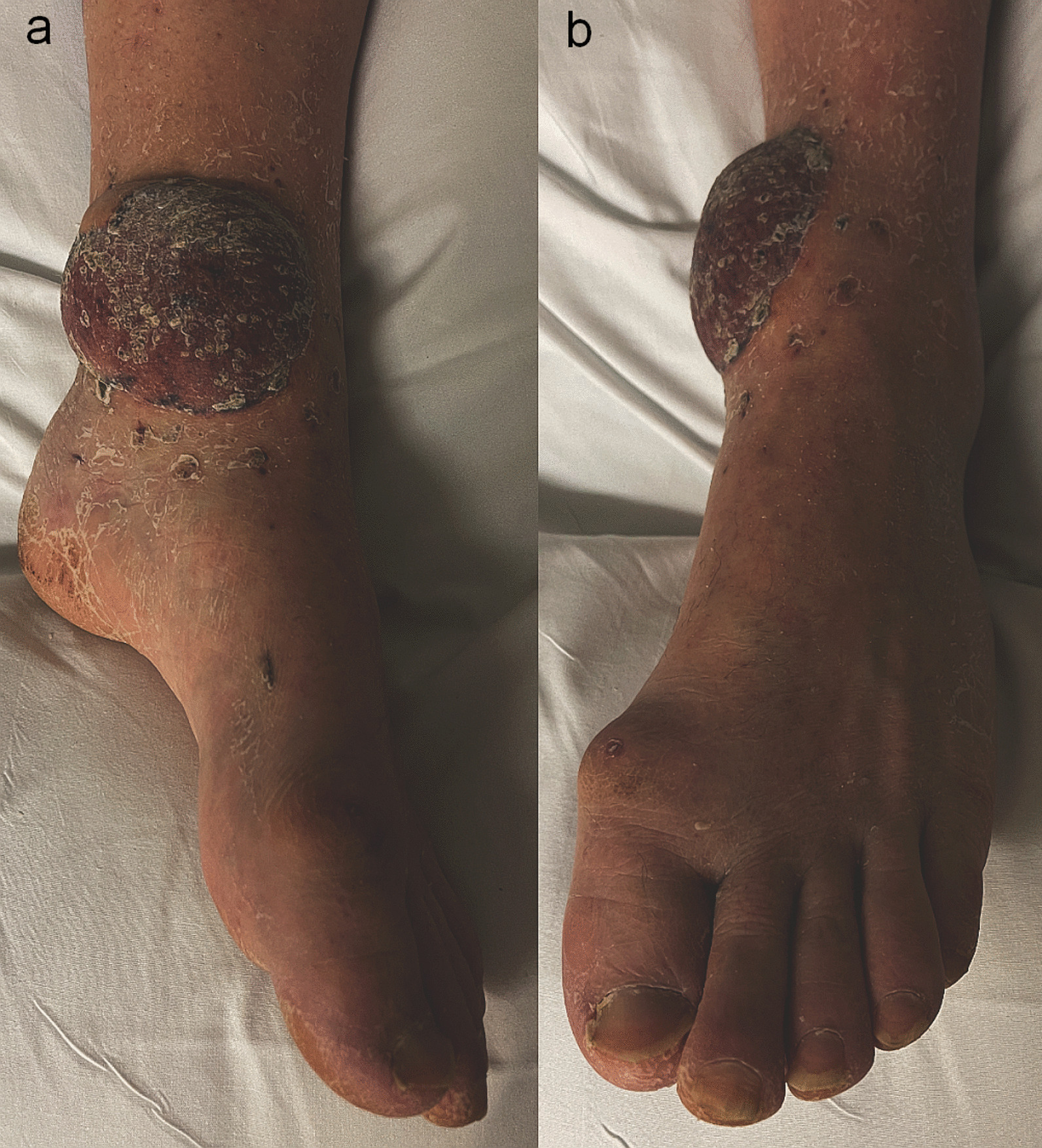
**a** and **b** Appearance of gracilis flap at left ankle at approximately 8 weeks from surgery, **a** medial view, **b** anterior view

A combined orthoplastic reconstruction of the left ankle was performed, with removal of the external fixator and internal fixation of the bimalleolar fracture and free gracilis flap reconstruction harvested from the right thigh. A palpable and Doppler-audible dorsalis pedis artery was present at the start of the case, but no signal was present proximal to the anterior ankle. During the preparation of the left ankle recipient vessels, it was noted that the PAM passed from lateral to medial across the posterior distal tibia in the coronal plane. It was accompanied by two venae comitantes on the medial and lateral aspect of the artery that were each of similar caliber to the artery itself. The diameter of the PAM at the posterior tibia just proximal to the medial malleolus was similar in size to that expected of a posterior tibial artery. Intraoperative microvascular clamping of the artery resulted in foot hypoperfusion, with skin pallor and capillary refill time of 4 seconds, however this gradually improved to 3 seconds with resolution of pallor over approximately 1 hour, despite the clamp being applied. An end-to-side anastomosis was performed using a circular arteriotomy of the PAM, and one venous anastomosis end-to-end with the medial vena comitans to the PAM. Two days after reconstruction, capillary refill returned to 1–2 seconds in the left foot, and the dorsalis pedis artery remained palpable. The healed left gracilis flap at 8 weeks is shown in Fig. 4a and b. Following microsurgical reconstruction, aspirin 75 mg OD and lansoprazole 30 mg OD were given for 6 weeks.

Postoperatively, he developed a cough and *Klebsiella aerogenes* was isolated from sputum. On the advice of the microbiology team, he was commenced on a single dose of gentamicin 480 mg intravenously, plus piperacillin and tazobactam 4.5 g intravenously TDS for 7 days and co-trimoxazole 960 mg intravenously BD for 6 weeks. The right foot plantar degloved flap subsequently developed full thickness necrosis, with 50% necrosis of abductor hallucis, and exposed calcaneum with a thick malodorous exudate. *Escherichia coli* was isolated from tissue cultures, urine, and blood cultures, with additional *Clostridium novyi* and *Enterococcus hirae* identified from right heel tissue cultures. Right foot debridement was performed, and topical vancomycin and tobramycin in a calcium sulphate carrier (Stimulan®, Biocomposites Ltd., Keele, England) was applied to the exposed bone, in conjunction with the above intravenous antibiotic therapy.

**Fig. 5:**
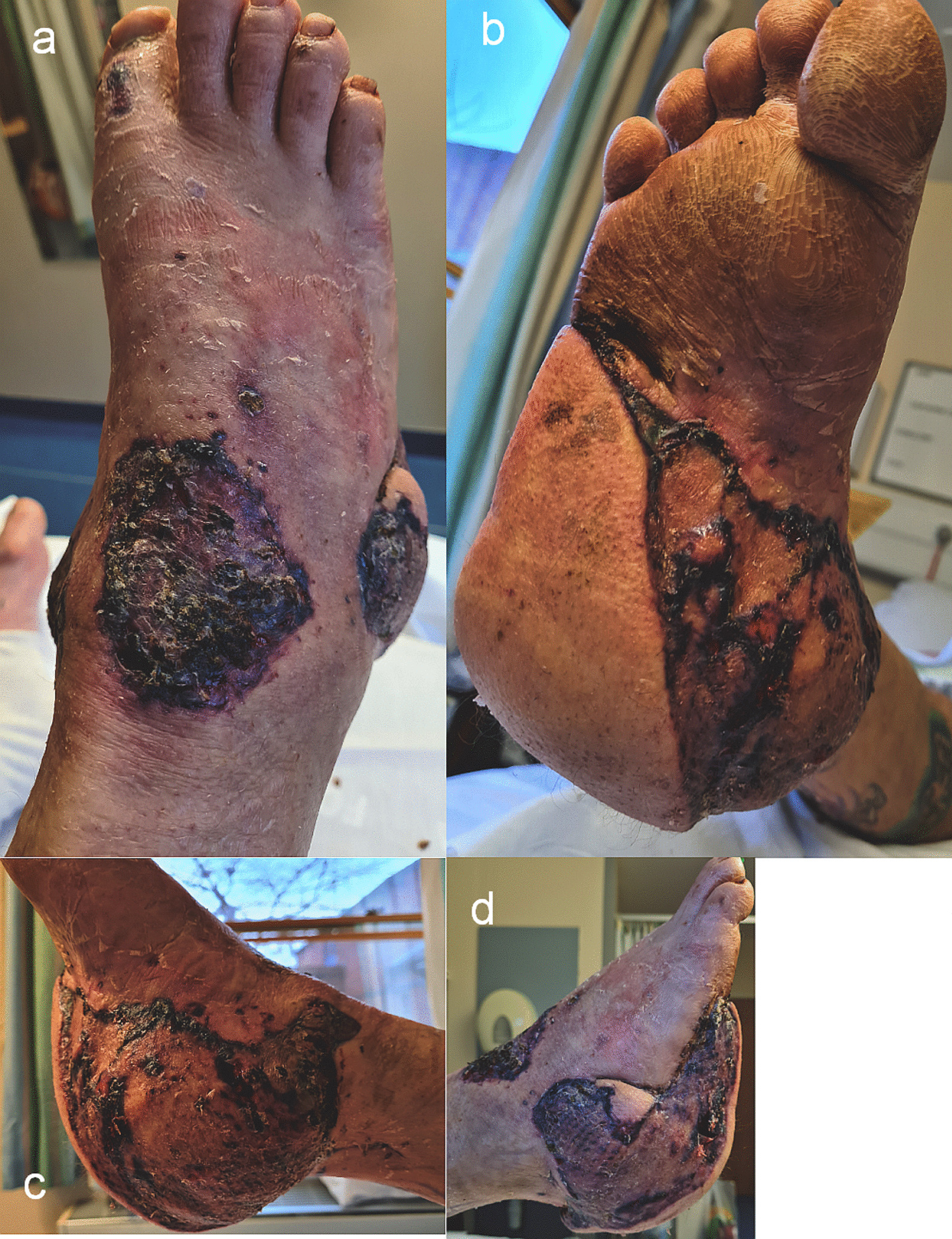
**a**–**d** Appearance of the right foot and ankle chimeric musculocutaneous latissimus dorsi—serratus anterior fascia flap at approximately 8 weeks from surgery, **a** dorsal, **b** plantar, **c** medial, **d** lateral

Following the above debridement, when the wounds were macroscopically clean, a final debridement and simultaneous chimeric free musculocutaneous latissimus dorsi-serratus fascial flap was performed for right foot and ankle reconstruction, via end-to-side microanastomosis to the right posterior tibial artery. The 8 week outcome is presented (Fig. 5a–d). The patient developed COVID-19 related pneumonia 2 weeks after his second free flap operation (positive COVID-19 nucleic acid polymerase chain reaction test from nasopharyngeal swab), from which he made a full recovery after an extended inpatient stay.

Other medications include laxatives, which were required for the second and third weeks of admission, namely Movicol (Norgine, Harefield, UK) 2 sachets PO TDS, senna 15 mg PO nocte (every night), followed by lactulose 15 ml PO BD until discharge. Zopiclone 3.75 mg PO PRN nocte was prescribed during the third inpatient month, and a protein nutritional supplement was commenced in the last 3 weeks of admission and continued after discharge.

**Fig. 6:**
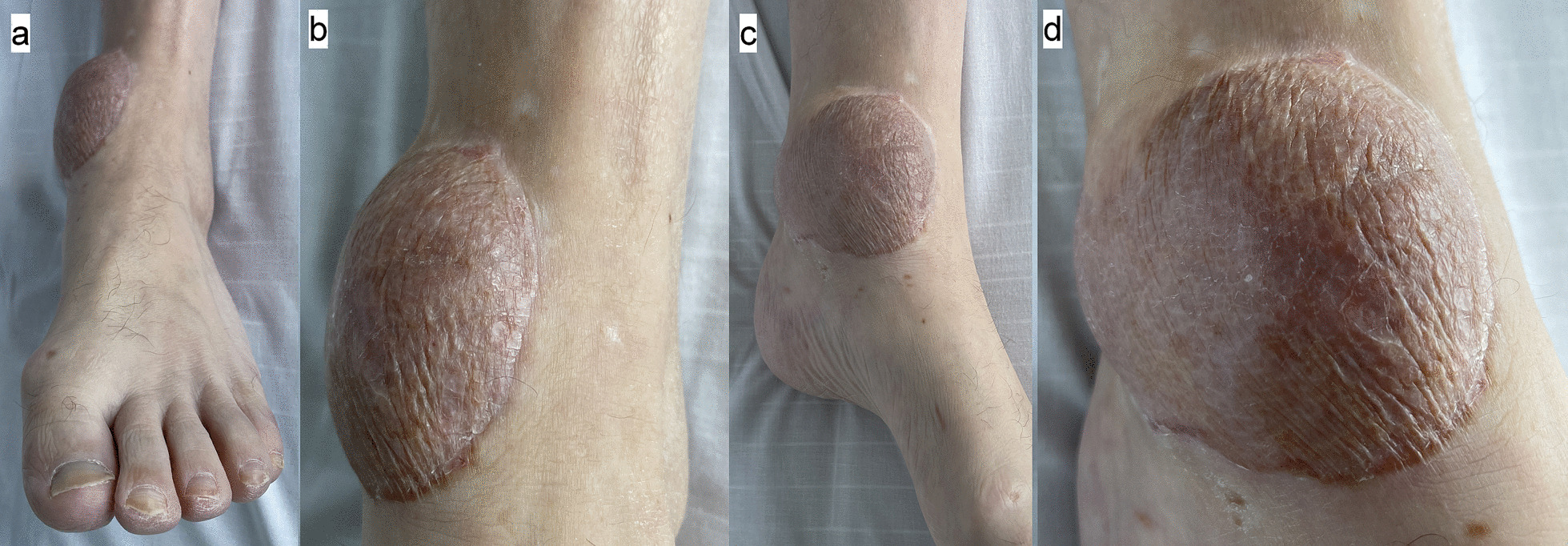
**a**–**d** Appearance of the left foot gracilis flap and split skin graft at left ankle at approximately 7 months from surgery. **a** Anterior view, **b** anterior view close-up of flap, **c** medial view, **d** medial view close-up of flap

Intensive rehabilitation and physiotherapy continued after the 3 months of inpatient hospital stay. The right lower limb flap developed skin graft loss and recurrent soft tissue infections with *Pseudomonas aeruginosa* at 4 months after surgery, requiring readmission and debridement. The radial nerve palsy had partially recovered at 6 months with active wrist extension present; nerve conduction studies and electromyography noted findings consistent with a severe axonotmesis lesion at the upper humerus level with features suggestive of reinnervation. The left ankle flap had a good outcome at 7 months with no evidence of deep infection (Fig. 6a–d).

## Discussion

This polytrauma case with a rare anomalous left lower limb peronea arteria magna in a limb that required microsurgical soft tissue reconstruction demonstrates that the use of this vessel is technically feasible in these circumstances and informs surgeons who encounter this anomaly of the potential reconstructive options and aspects of the surgical technique suggested. Free tissue transfer onto this vessel has rarely been described, and only with radiographic collaterals, which were absent in this case.

**Fig. 7:**
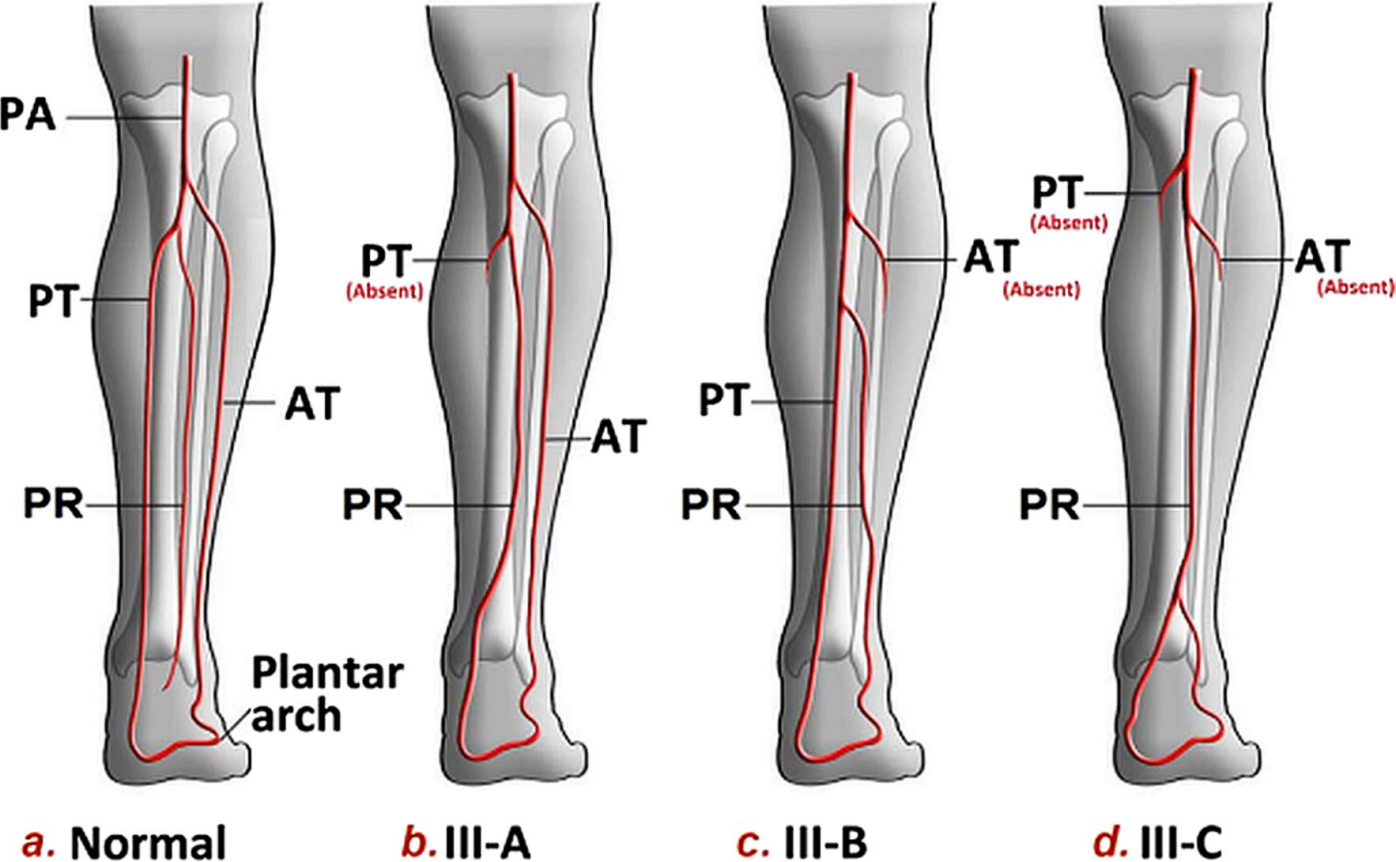
Normal and dominant peroneal artery infra-popliteal branching patterns according to the unified classification (III A-C patterns). *PA* popliteal artery, *PT* posterior tibial artery, *AT* anterior tibial artery, *PR* peroneal artery. Reproduced with permission, Abou-Foul and Borumandi, Microsurgery, 2016. Oxford University Press [1]

Free flap reconstruction of the lower limb in trauma requires careful assessment of the appropriate recipient vessels and zone of trauma. Potential arterial recipient vessels include anterior or posterior tibial and peroneal or dorsalis pedis. Recipient veins include the venae comitantes, one vena comitans and the long saphenous, or the long saphenous alone [5]. Contrast vascular imaging may reveal occult arterial injuries, or anatomical variants such as the PAM, as in this case. Lutz *et al.* reported successful foot and ankle free flap reconstruction utilizing the PAM as the recipient vessel with an end-to-side arterial microanastomosis, but reported that sufficient collateral perfusion to the foot was present on angiography and also after arterial clamping [6]. Balloon occlusion angiograms have been used to assess foot perfusion in PAM cases for planned free fibula harvest [7]. Successful harvest of a free fibula flap in a patient with Kim–Lippert popliteal branching pattern IIIB has been reported [8], although the case we present is perhaps closest to IIIC [1], as shown in Fig. 7.

In the IIIC variation, the plantar arch is usually supplied by the PAM traversing from lateral to medial, posterior to the tibia in the distal third of the leg, then entering the tarsal tunnel akin to the distal posterior tibial artery (proximal to the origin of the medial plantar artery). This was evident in the preoperative imaging in the case presented, prompting a surgical approach to access the recipient vessels immediately proximal to the tarsal tunnel to perform the end-to-side anastomosis.

The venae comitantes (vc) in the PAM were notably different to those typically associated with a posterior tibial artery (PTA) [9]. Compared with usual vc-PTA-vc anteroposterior sagittal orientation of the posterior tibial artery (anterior to the tibial nerve), the orientation may be vc-PAM-vc in the coronal plane, and the PAM may lie anterior or posterior to the tibial nerve. This coronal vc-PAM-vc arrangement was present in our case and is consistent with the description of the anatomy of the PAM in a previous cadaveric report [10]. Awareness of this possible local variation is important for the surgeon to select the best site for flap microanastomosis. Knowledge of PAM vessel configuration also helps the surgeon to correctly identify that the most superficial (medial) vessel is a PAM vena comitans despite visible transmitted pulsations and that the lateral (deeper) PAM vena comitans may be difficult to access as a recipient vein.

## Conclusion

We report that successful free flap reconstruction may be performed to a lower limb with a peronea arteria magna recipient as the lone vessel supplying the foot in trauma, although preoperative counseling of the risks, benefits, and options are essential.

## Data Availability

All data in this case report are available from the corresponding author on reasonable request.

## Abbreviations

AT: Anterior tibial artery
BD: Twice daily
COVID-19: Coronavirus disease 2019
CT: Computed tomography
IM: Intramuscular
OD: Once daily
PA: Popliteal artery
PAM: Peronea arteria magna
PO: Per orem (by mouth)
PR: Peroneal artery
PRN: Pro re nata (as needed)
PT: Posterior tibial artery
QDS: Four times daily
SC: Subcutaneous
TNPWT: Topical negative pressure wound therapy
TDS: Three times daily
vc: Vena comitans

## References

[1] Abou-Foul AK, Borumandi F (2016). Anatomical variants of lower limb vasculature and implications for free fibula flap: systematic review and critical analysis. Microsurgery.

[2] Kim D, Orron D, Skillman JJ (1989). Surgical significance of popliteal arterial variants. A unified angiographic classification. Ann Surg.

[3] Lippert H, Pabst R (1985). Arterial variations in man: classification and frequency.

[4] Rosson G, Singh N (2005). Devascularizing complications of free fibula harvest: peronea arteria magna. J Reconstr Microsurg.

[5] Cho EH, Garcia RM, Pien I, Kuchibhatla M, Levinson H, Erdmann D, Levin LS, Hollenbeck ST (2016). Vascular considerations in foot and ankle free tissue transfer: analysis of 231 free flaps. Microsurgery.

[6] Lutz BS, Siemers F, Shen Z-L, Machens H-G, Wippermann B, Berger A (2000). Free flap to the arteria peronea magna for lower limb salvage. Plast Reconstr Surg.

[7] Rahmel BB, Snow TM, Batstone MD (2011). Fibular free flap with arteria peronea magna: the role of preoperative balloon occlusion. J Reconstr Microsurg.

[8] Betar NM, Subramaniam SS, Borgna SC (2020). Fibula free flap with type IIIB popliteal artery branching: a case report and recommendations. Eur J Plast Surg.

[9] Bulla A, Bolletta A, Fiorot L, Maffei M, Bandiera P, Casoli V, Montella A, Campus GV (2019). Posterior tibial perforators relationship with superficial nerves and veins: a cadaver study. Microsurgery.

[10] Jung W, Oh CS, Won HS, Chung IH (2008). Unilateral arteria peronea magna associated with bilateral replaced dorsalis pedis arteries. Surg Radiol Anat.

